# Genetically engineered bacteriophages as novel nanomaterials: applications beyond antimicrobial agents

**DOI:** 10.3389/fbioe.2024.1319830

**Published:** 2024-04-25

**Authors:** Seong-Min Kim, Hye Ryoung Heo, Chang Sup Kim, Hwa Hui Shin

**Affiliations:** ^1^ Medical Device Development Center, Daegu-Gyeongbuk Medical Innovation Foundation, Daegu, Republic of Korea; ^2^ Department of Chemical and Biochemical Engineering, Dongguk University, Seoul, Republic of Korea

**Keywords:** bacteriophage, nanomaterial, biosensor, tissue regeneration, genetic engineering, filamentous phage, M13, phage display

## Abstract

Bacteriophages, also known as phages, are viruses that replicate in bacteria and archaea. Phages were initially discovered as antimicrobial agents, and they have been used as therapeutic agents for bacterial infection in a process known as “phage therapy.” Recently, phages have been investigated as functional nanomaterials in a variety of areas, as they can function not only as therapeutic agents but also as biosensors and tissue regenerative materials. Phages are nontoxic to humans, and they possess self-assembled nanostructures and functional properties. Additionally, phages can be easily genetically modified to display specific peptides or to screen for functional peptides via phage display. Here, we demonstrated the application of phage nanomaterials in the context of tissue engineering, sensing, and probing.

## 1 Introduction

The discovery and manipulation of novel nanomaterials in materials science and biotechnology has sparked innovative avenues for research and development. Among these emerging nanomaterials, genetically engineered bacteriophages are gaining prominence as versatile and multifunctional entities that far exceed their classical role as antimicrobial agents.

Bacteriophages, also known as phages, are viruses that possess a remarkable specificity for infecting bacteria. Each type of bacteriophage tends to target a specific species or even strain, thus making them valuable tools for selectively combating harmful bacteria, including antibiotic-resistant strains, in a process known as “phage therapy” (Kortright et al., 2019; Pirnay, 2020).

Traditionally, bacteriophages have been primarily employed based on their antimicrobial properties to target and eliminate pathogenic bacteria in a variety of environments, including clinical and food safety applications (Sunderland et al., 2017; Kortright et al., 2019). However, recent scientific interest in phages has increased due to their exceptional characteristics. These viruses exhibit distinctive structural features. Some such as filamentous phages possess an elongated, filament-like structure, while others may exhibit polyhedral or complex shapes. These structural differences have been exploited for various purposes, including nanomaterial engineering and drug delivery (Chung et al., 2011; Lee et al., 2012; Sunderland et al., 2017).

One of the most notable characteristics of bacteriophages is their genetic malleability. Advances in genetic engineering techniques have unlocked numerous possibilities that have enabled researchers to reprogram phages at the genetic level. This transformative capacity allows researchers to customize phages to perform various tasks by modifying their genetic material to encode specific surface proteins or peptides. This genetic engineering capacity has opened a wide range of possibilities that include creating phage-based nanomaterials and tailoring phages for targeted tissue regeneration and diagnostic applications.

Phage display is a technique that is used for evolutionary screening using filamentous phage libraries and has contributed to the discovery of selective functional peptide ligands (Smith, 1985; Smith and Petrenko, 1997; Barderas and Benito-Peña, 2019). These ligands can be precisely customized to interact with specific target molecules or surfaces, thereby expanding the potential of bacteriophages for use in nanotechnology and biomedical engineering.

This study explored genetically engineered bacteriophages as novel nanomaterials by delving into their unique attributes and the diverse landscape of applications that they unlock. In particular, we aimed to highlight the potential and impact of phages as novel materials by providing insights into the basis of bacteriophages, examples of their application in the field of tissue regeneration as nanomaterials, and research cases involving various types of biosensors.

## 2 What is a bacteriophage?

### 2.1 Bacteriophage

**FIGURE 1:**
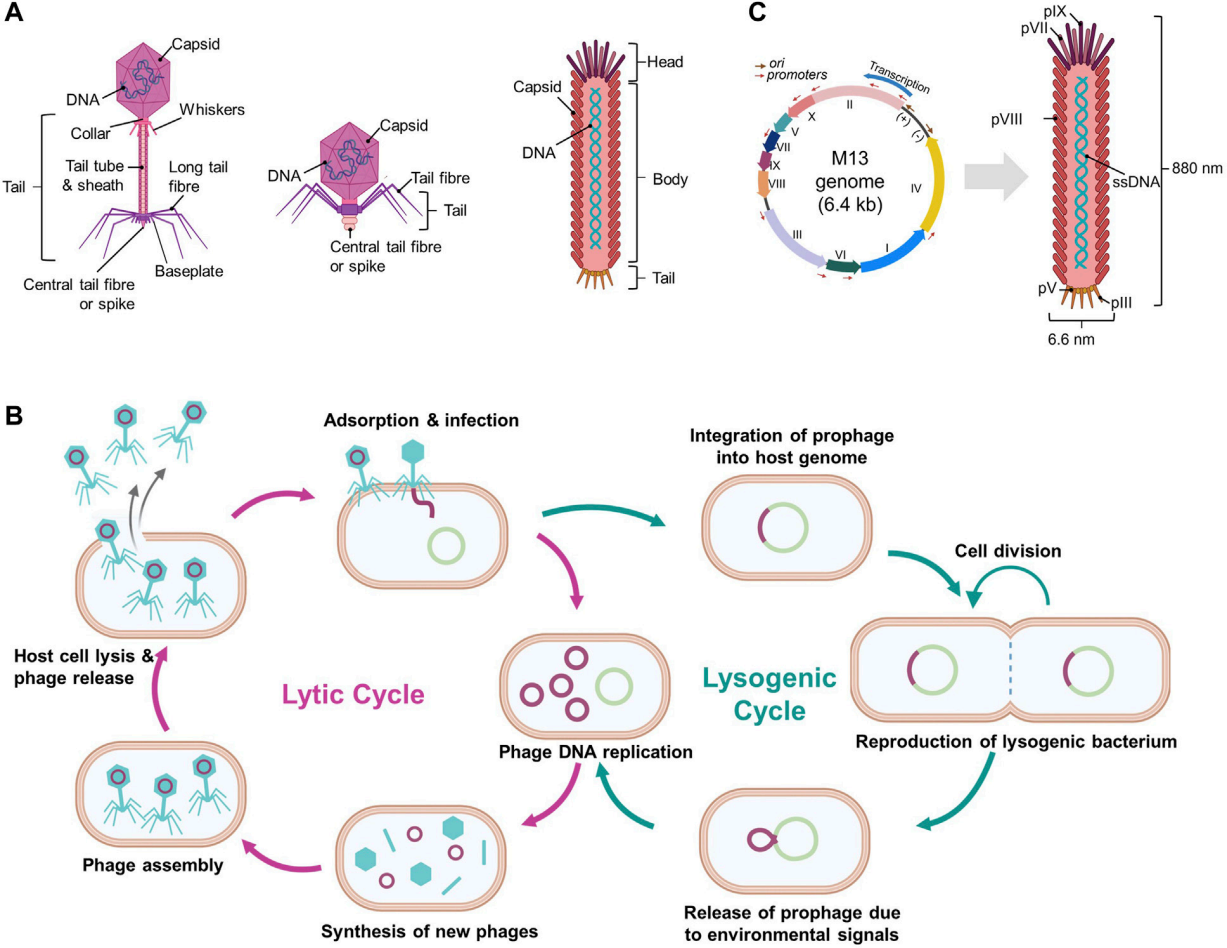
Schematic diagrams of bacteriophages and their life cycles. **(A)** Various structures of bacteriophages, **(B)** Overview of the phage life cycles: lytic and lysogenic, and **(C)** Filamentous M13 phage and its genome. (Created with Biorender.com. (B) was adapted from “Lytic and Lysogenic Cycle”, by BioRender [2023]).

Bacteriophages are viruses that replicate in bacteria. Bacteriophages possess DNA or RNA genomes encapsulated by proteins such as capsid proteins. Its genome can replicate in bacteria after injection into bacterial cells and subsequent use of the bacterial replication machinery. There are numerous bacteriophages of various sizes and shapes, and phages can be simple or highly sophisticated, although all are typically composed of “head,” “body,” and “tail” regions (Figure 1A).

Bacteriophages are host-dependent and typically infect microbial species or even certain strains below a species. When bacteriophages attach to host cells, they initiate either a lytic or a lysogenic replication cycle (Figure 1B) (Hobbs and Abedon 2016; Popescu et al., 2021). Certain bacteriophages exhibit a pseudo-lysogenic cycle that is defined as bacteriophages lacking either genome replication such as in the lytic cycle or genome integration such as in the lysogenic cycle, and this allows for the presence of a phage genome inside the host cell without any degradation (Hobbs and Abedon 2016; Doss et al., 2017).

#### 2.1.1 Lytic phage (virulent phage)

Lytic pages, including T1-T7 phages, typically consist of a head and a flexible tail but lack the shape of long filaments (Hobbs and Abedon 2016). Once a lytic phage adheres to a susceptible bacterial cell, it injects its genome into the cytoplasm of the host. The host replication machinery duplicates the phage genome and expresses the encoded proteins such as capsid proteins. The phage genome and proteins in the host cell are assembled into particles. When phage particles reach maturity, the bacterial cells are either actively or passively lysed, thus releasing new phages and reinfecting other bacterial cells (Figure 1B). This process is rapid and requires only a few minutes to complete (Hobbs and Abedon 2016; Doss et al., 2017).

#### 2.1.2 Lysogenic phage (temperate phage)

In contrast to lytic phages, lysogenic phages (also known as temperate phages) do not cause immediate lysis of host cells. Lysogenic phages initiate infections by attaching to and injecting their genomes into susceptible bacterial cells. The injected genome is typically integrated into the bacterial cell genome (known as a prophage) or maintained as plasmid DNA and thus replicated and inherited by daughter bacterial cells without harming the host cells (Figure 1B) (Hobbs and Abedon 2016; Popescu et al., 2021). The lysogenic cycle can become a lytic cycle depending on the conditions of the host cells such as nutrient depletion (Erez et al., 2017). Lambda phages are well known for being lysogenic and following lytic cycles, and a group of filamentous phages, including M13, fd, Ff, and F1 phages, also belong to lysogenic phages (Loh, Kuhn, and Leptihn 2019; Howard-Varona et al., 2017).

### 2.2 Filamentous phage, M13

Filamentous phages are among the simplest organisms and possess a long, flexible, fiber-like structure with an approximate diameter of 6 nm and a length ranging from 1000 to 2000 nm. Some well-known filamentous phages such as M13, fd, and f1 belong to the “Ff” group within the *Inovirus* genus, as they share significant similarities with each other (Knezevic et al., 2021).

The M13 phage is composed of circular single-stranded DNA (ssDNA) encapsulated with approximately 2700 major coat proteins called pVIII and other minor coat proteins such as pIII, pVI, pVII, and pIX (Figure 1C) (Kehoe and Kay, 2005). At each end of the M13 phage, there are two minor coat proteins, and five copies each of pVII and pIX are located at one end and five copies each of pIII and pVI are present at the other end (Barbas et al., 2001). The M13 phage genome is approximately 6407 base pairs long and encodes 10 genes, including those encoding coat proteins (Figure 1C) (Kehoe and Kay, 2005; Chung et al., 2011). Phage length depends upon the length of its genomic ssDNA, and genetically engineered M13 phages can vary in length (Kehoe and Kay, 2005). Interestingly, up to 12,000 bases can be inserted into the genome of a wild-type phage without affecting the virion packaging (Marvin 1998).

The M13 phage infects host bacteria possessing an F pilus such as *Escherichia coli*. The infection and replication processes commence when the pIII coat proteins of M13 bind to the F pilus. After this binding, the genome of M13 that encodes 11 proteins is transferred into the host cell, and the M13 phage disassembles. Once the M13 genome enters a bacterial cell, the genomic ssDNA is converted into double-stranded DNA (dsDNA), and the encoded proteins are synthesized using bacterial machinery (Smeal et al., 2017).

Virion assembly is triggered when ssDNA and expressed proteins of the M13 phage accumulate inside the infected bacteria. Upon synthesis, the coat proteins pVIII, pVII, pIX, pVI, and pIII spontaneously insert into the inner bacterial membrane and await ssDNA replication (Barbas et al., 2001). When the pV concentration is sufficient, it coats newly replicated ssDNA genomes and prevents their conversion to dsDNA. As ssDNA that forms an imperfect hairpin is free of pV and is captured by a complex of integral membrane proteins pVI, pXI, and pI, the virion is assembled and simultaneously extruded from the membrane pores of the bacteria (Smeal et al., 2017; Barbas et al., 2001; Kehoe and Kay, 2005). During assembly, pV is separated from the phage ssDNA genome, and the phage genome is coated with pVIII. Five copies each of pVII and pIX cover the end of the ssDNA-containing virion and form an incomplete hairpin (Kehoe and Kay, 2005; Smeal et al., 2017). While the phage virion was released from the bacterial cells, five copies each of pIII and pVI are sealed at opposite ends. Additionally, pIII assists phage secretion from host bacteria. As M13 is a nonlytic bacteriophage, host cells are not killed by the infection. Infected bacterial cells continue to grow and divide indefinitely; however, they grow at a slower rate than do the uninfected bacteria (Barbas et al., 2001; Smeal et al., 2017).

### 2.3 Phage therapy: conventional application of phages

When phages were discovered by Ernest Hanbury Hankin in 1896, they were reported to act as antimicrobials in the waters of the Ganges River in India (Hankin, 1896). Phages were also independently identified as small agents that infect and kill bacteria by Twort in 1915 (Twort, 1915) and by d’Hérelle in 1917 (d’Herelle, 1917). This intrinsic antibiotic activity has led to the study and the development of “phage therapy” in which phages or phage cocktails are administrated to patients suffering from bacterial infections. Additionally, phages activate the human immune system to induce inflammation caused by lysis of bacterial cells. Thus, phages can directly kill and eliminate bacteria and support the human immune system by combating bacteria (Reardon, 2014; Doss et al., 2017; Sunderland et al., 2017).

Although phage therapy was implemented in Eastern Europe and the Soviet Union in the 1900s, the use of phages as antimicrobial agents has not been established in Western Europe or the United States. Phage therapy is still widely used in Russia, Georgia, and Poland, but it is not used elsewhere, as Eastern European studies focused on medical efficacy and safety do not meet Western standards (Reardon, 2014). Nevertheless, phage therapy has recently attracted attention due to the emergence of multidrug-resistant bacteria caused by the over-use of antibiotics. Antibiotics work against a wide range of bacteria, including non-pathogenic bacteria and gut microbes. However, phages treat only one specific species or strain. Additionally, phage therapy can reduce side effects, as phages do not infect the human body, and resistance is difficult to develop. Despite several advantages, there is still room for improvement in phage therapy such as identifying more effective phages for certain infections, creating phage cocktails with higher therapeutic potential, and engineering phages to achieve multi-host specificity (Pires et al., 2016; Kortright et al., 2019).

### 2.4 Genetically engineered phages

**FIGURE 2:**
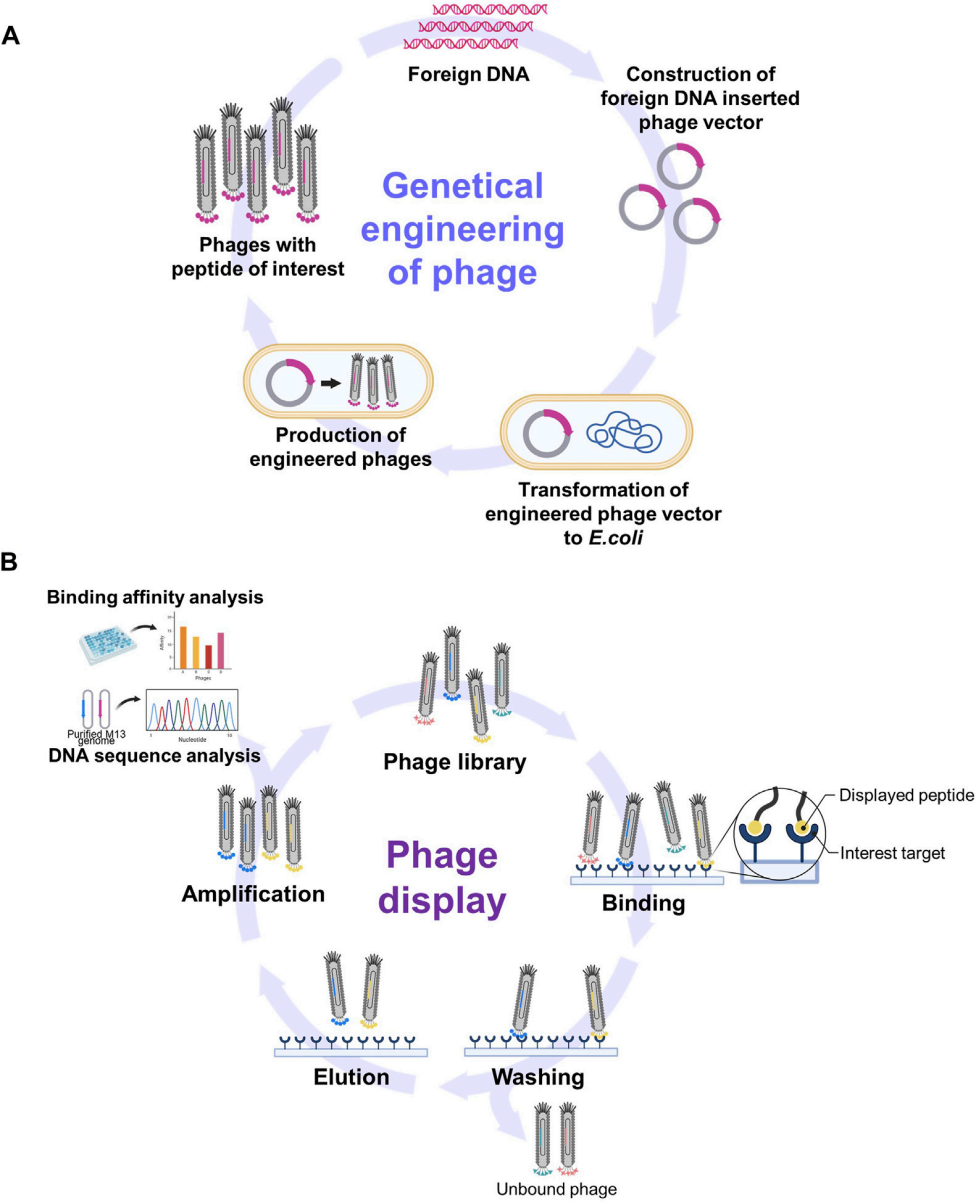
Schematic diagrams of phage genetic engineering and phage display. **(A)** The process of incorporating foreign peptides or proteins into phages through genetic engineering. **(B)** The summary of phage display process for screening functional epitopes. (Created with Biorender.com. [B] was adapted from “Phage Display Panning”, by BioRender [2023]).

Genetic engineering involves altering the genetic material of an organism to introduce novel traits or functions. In the context of the M13 phage, these manipulations typically revolve around modifying its DNA to express specific proteins such as antibody fragments or peptides. This was achieved by inserting foreign DNA sequences into the phage genome that could then be replicated and expressed along with phage genes (Figure 2A).

The M13 phage is considered to be a well-constructed genetic engineering system for several reasons. First, M13 possesses a relatively small and simple genome with well-characterized gene sequences, thus making it easier to modify and manipulate. In addition, M13 phages can serve as cloning vectors to introduce DNA fragments of interest. This system allows for the insertion of foreign DNA into the phage genome that can then be replicated and packaged into new phage particles (Chung et al., 2011). Second, concepts such as phage display are well defined in relation to the expression of foreign peptides or proteins on the surface of the M13 phage. This is achieved by incorporating foreign peptides or proteins into coat proteins such as pIII and pVIII, thereby easily adding novel functionality (Smith and Petrenko, 1997; Barbas et al., 2001; Kehoe and Kay, 2005). Finally, the production of engineered M13 phages is straightforward due to their nonlytic nature where they do not immediately destroy host cells upon infection (Barbas et al., 2001; Kehoe and Kay, 2005). Furthermore, the lifecycle of the M13 phage within host cells, including its infection and replication mechanisms, has been well studied and understood (Kehoe and Kay, 2005). This knowledge will facilitate the manipulation of various stages of infection, including DNA replication, protein expression, and phage assembly.

### 2.5 Phage display for screening functional epitopes

Phage display has emerged as a versatile and indispensable technique in molecular biology and biotechnology. George P. Smith, the winner of the 2018 Nobel Prize in Chemistry, first reported this technique in 1985 (Smith and Petrenko, 1997; Barderas and Benito-Peña, 2019). The basis of phage display is the genetic fusion of random foreign sequences with phage DNA. This process produces encoded peptides on the surface of the phage virions, collectively called phage libraries. This display allows for a direct correlation between genotype and phenotype, thus providing unique insights into protein structure, function, and interactions.

Based on the site of fusion on M13, several types of phage libraries have been developed, including the pIII library where foreign peptides are fused to the N-terminus of pIII, the pVIII library (commercially available from NEB Inc.)(Smith and Petrenko, 1997; Hoess, 2001; NEB, 2002; Kehoe and Kay, 2005) where fusion occurs at the N-terminus of pVIII (Petrenko et al., 2002; Fagerlund et al., 2008; Kuzmicheva et al., 2009), and the pVI library that involves fusion to pVI (Hufton et al., 1999). The genetic diversity inherent in a phage library that encompasses a broad spectrum of peptide variants enables the screening of extensive peptide landscapes, thereby facilitating the identification of candidates with the desired properties.

The exposure of phages carrying diverse inserts to a target substance provides an opportunity to navigate expansive libraries in the pursuit of peptides or even proteins that interact with the target. The process of biopanning facilitates the selection of specific peptides or proteins exhibiting affinity for a given target (Figure 2B) (Smith and Petrenko, 1997; Barbas et al., 2001; Hoess, 2001; Kehoe and Kay, 2005). Biopanning encompasses four key stages: 1) the establishment of phage display libraries; 2) the capture stage where virions display sequences with an affinity for the target adhering to it; 3) the washing stage using a detergent-supplemented buffer to eliminate unbound virions; 4) the elution stage employing an acidic buffer (pH −2.2) that enables the collection of phages with precise affinity. Subsequently, the eluted phages are amplified via infection by host bacteria. This leads to the multiplication of phages, ultimately giving rise to a newly enriched pool (also known as a sub-library) with markedly reduced diversity that is primed for new interactions with the target. This iterative selection process recurs through multiple rounds until only a small number of peptide sequences emerge as prominently yielded (Barbas et al., 2001). These peptides are designated as target-specific peptides.

The versatility of phage display has revolutionized various fields ranging from antibody discovery (Parmley and Smith, 1988; Fellouse et al., 2007; Thie et al., 2009; Jones et al., 2010) to protein-protein interaction studies (Fuh et al., 2000; Hertveldt et al., 2009). Phage displays enable rapid antibody identification with the desired binding properties and contribute to deciphering protein networks and molecular interactions, thus leading to advancements in diagnostics (Kirsch et al., 2008; Deutscher, 2010; Kuhn et al., 2016; Li et al., 2023), therapeutics (Ladner et al., 2004; Saw and Song, 2019; Daly et al., 2023), and vaccine development (Gnanasekar et al., 2004; Wang and Yu, 2005; Aghebati-Maleki et al., 2016; Solís-Lucero et al., 2016; Stern et al., 2019; Staquicini et al., 2021; Grabowski et al., 2023). Phage displays also hold promise for applications in nanotechnology and biomaterials. For example, phage display-selected peptides demonstrate a penchant for binding to diverse nanoparticles such as ZnO (Golec et al., 2016) and Fe_3_O_4_ (Rawlings et al., 2015; You et al., 2016) and can also act as biosensors for detecting explosives or pathogens (Jaworski et al., 2008; Gutés et al., 2013; Rawlings et al., 2015; Peng and Chen, 2019; Wang et al., 2021). In the field of tissue engineering, this technique guides researchers in identifying peptides such as arginylglycylaspartic acid (RGD) by using phage display libraries (Pasqualini et al., 1997). These peptides have an affinity for specific cell types (Kelly and Jones, 2003; Qin et al., 2011; Asar et al., 2020), tissues, or organs (Pasqualini and Ruoslahti, 1996; Laakkonen et al., 2008; Li et al., 2011; Pleiko et al., 2021). The integration of these peptides into scaffolds or biomaterials orchestrates processes such as cell attachment, proliferation, and differentiation, thus eventually improving tissue regeneration (Martins et al., 2016).

## 3 Applications of genetically engineered phages as nanomaterials

From a materials science perspective, phages have emerged as robust building blocks of nanomaterials (−900 nm in length and −8 nm in width) that possess distinctive physical and chemical properties, thus making them well-suited for highly ordered assemblies with tailored properties (Cao and Mao, 2009). Furthermore, phages exhibit significant potential for use as biomaterials in biomedicine. These prospects are rooted in several key factors. 1) Through genetic engineering, one or more foreign peptides can be easily incorporated onto the surface of a single phage, thus facilitating the construction of multifunctional phage-based nanomaterials (Chung et al., 2011). 2) The phage display technique is a valuable tool for the selective identification of peptides with target-specific binding capabilities, thereby endowing nanomaterials with the desired characteristics (Kehoe and Kay, 2005; Chung et al., 2011). 3) It is worth noting that phages naturally inhabit the human body, particularly the human microbiota and bladder (Li et al., 2014; Miller-Ensminger et al., 2018). 4) Phages are biocompatible, biodegradable, and stable under physiological conditions. Multiple research groups have administered phage libraries (up to 10^14^ phage particles) to patients and conducted *in vivo* bio-panning to identify tissue-specific peptides without noticeable side effects. This highlights their excellent tolerance in humans (Arap et al., 2002; Krag et al., 2006; Shukla et al., 2013; Christiansen et al., 2015). 5) Phages possess the unique ability to form highly ordered structures such as liquid crystals at certain concentrations (Sawada, 2017). This property makes phages exceptional biomaterials for self-assembly into three-dimensional scaffolds. This versatility enables phages to function as nanobiomaterials and scaffolds for a wide range of applications, including biosensors (Park et al., 2015; Lee et al., 2017; Moon and Kim, 2017), tissue engineering (Merzlyak et al., 2009; Cao et al., 2019; Chang et al., 2023), and drug delivery systems (Ghosh et al., 2012; Karimi et al., 2016; Ju and Sun, 2017).

### 3.1 Phages for tissue regeneration

Tissue regeneration refers to the restoration of tissue damaged by accident or disease to a state where it can function normally (Boisseau and Loubaton, 2011; Cao et al., 2019). Research focused on tissue engineering has been actively conducted to aid in regeneration using tissue engineering technology when damaged tissue is difficult to heal naturally or when a large part of an organ is lost. Many researchers have studied biomaterials and their applications in the context of tissue regeneration. For example, a scaffold using an extracellular matrix (ECM)-based artificial skin helps to regenerate burned skin (Abdulghani and Mitchell, 2019; Gaharwar et al., 2020) and hydroxyapatite is used to fill physically damaged bones (Kattimani et al., 2016; Ramesh et al., 2018).

Materials were limited to target cells and/or certain biomaterials in early tissue engineering; however, recently, many researchers have widened the range of materials that have been used for this process, including cultured cells or supplemental materials (e.g., growth factors or cytokines) (Abdulghani and Mitchell, 2019). Bacteriophages and genetic engineering are promising materials for tissue engineering. This chapter describes recently published studies that have used bacteriophages as materials for tissue engineering.

#### 3.1.1 Advantage of phages in regeneration

**FIGURE 3:**
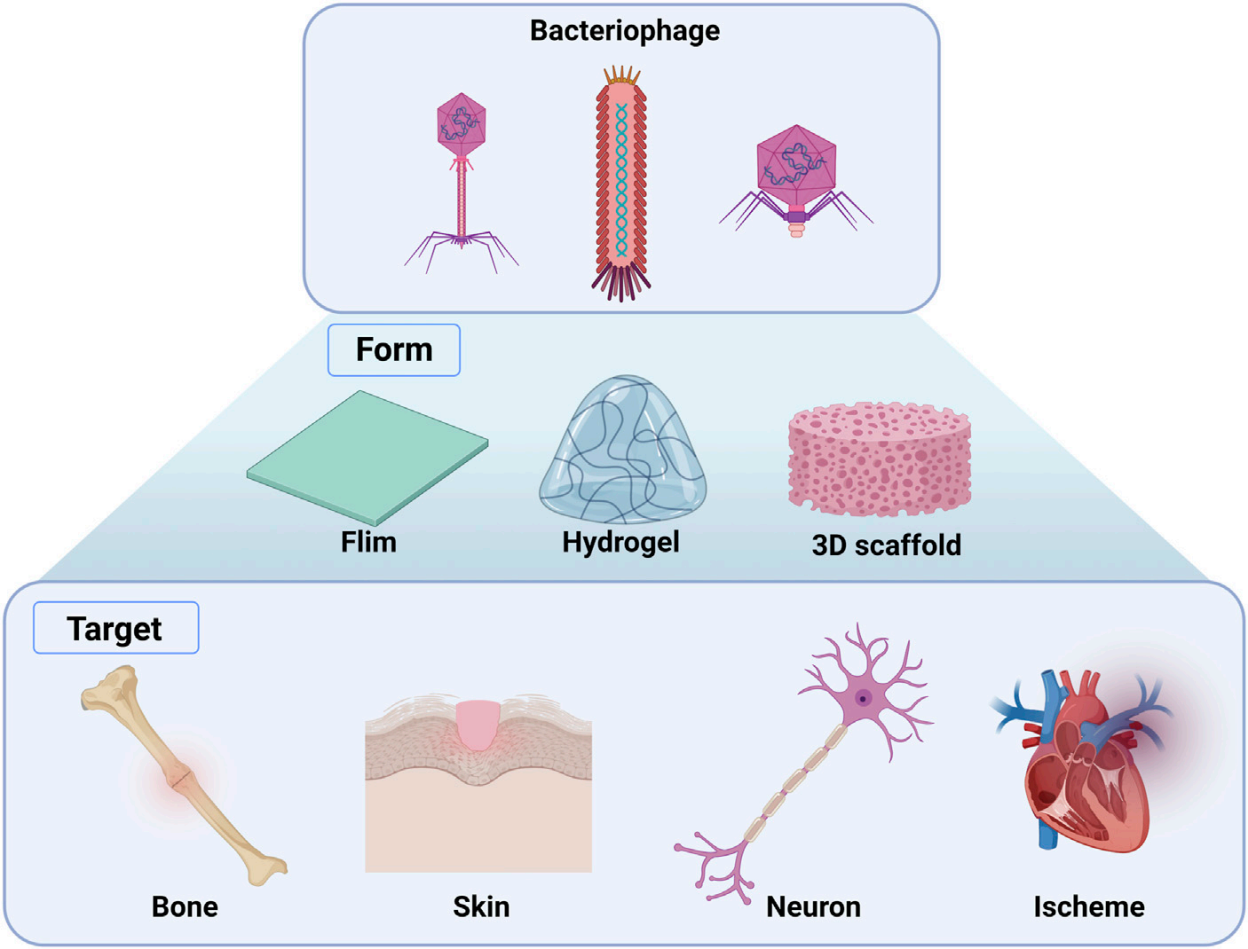
Overview of the applications of genetically engineered bacteriophages in tissue regeneration. Films, hydrogels, and 3D scaffolds can be fabricated using genetically engineered phage-targeting tissues, including bone, skin, neurons, and schemes. (Created with Biorder.com).

Bacteriophages are bacteria-specific and human-safe viruses possessing biological nanostructures composed of nucleic acids and proteins. In addition, they self-assemble when inserted into or injected into the body (Cao et al., 2016). Using genetic engineering technologies, bacteriophages can express functional peptides or proteins on their coat proteins to 1) contribute to cell proliferation, differentiation, and migration, 2) use the affinity of phage surface peptides for targeting specific cells or tissues, and 3) deliver a specific gene to the target tissue to alter the cell state to induce stem cell differentiation (Cao et al., 2016; Lee and Warner, 2017; Yue et al., 2022). Therefore, genetically engineered phages have received considerable attention as biomaterials for use in tissue engineering (Figure 3; Table 1).

**TABLE 1:** Engineered phages for tissue regeneration.

Target	Phage	Display site	Sequences	Materials	Loaded cells	Functions	Form	Reference
**Bone**	M13	pVIII	RGD	HA/β-TCP, chitosan	MSCs	Osteogenesis, angiogenesis	3D scaffold	Wang et al. (2014)
	M13	pVIII (M13KE)	RGD	Alg, PCL	-	Osteogenesis	3D scaffold	Lee et al. (2016)
	-	-	DPI-VTK	Bone-like minerals (calcium, phosphate)	iPSC-MSCs	Osteogenesis, angiogenesis	3D scaffold	Ramaraju and Kohn (2019)
	LM99	-	-	Alg, nanoHA	-	Osteogenesis, anti-infection	Hydrogel	Barros et al. (2020)
**Skin**	M13	pVIII (M13KE)	RGD, IKVAV	-	NIH-3T3	regeneration	Film	Jin and Lee (2018)
	T7	-	-	Gelatine, oxalginate, HA, aFGF	-	Wound healing, anti-infection	Hydrogel	Zhang et al. (2020)
	JD007	-	-	Ce6, MnO_2_ (Additional: NIR laser)	-	Wound healing, anti-infection	Solution	Wang et al. (2023)
	M13		Pol-K	AuNR, Zn^2+^ (Additional: NIR laser)	-	Wound healing, anti-infection	Solution	Peng et al. (2022)
**Neuron**	M13	pVIII	WT, RGD	-	NSC (derived from iPSC)	Neuro-differentiation	Film	Zhou et al. (2019)
**Ischemia**	M13	pVIII	RGD	PA	EPC	Angiogenesis	Hydrogel	Shrestha et al. (2022)

#### 3.1.2 Phages for bone regeneration

Bioengineers have used biocompatible materials and stem cells such as pre-osteoblasts, mesenchymal stem cells (MSCs), and induced pluripotent stem cells (iPSCs) for bone regeneration. For this process, the most popular material is hydroxyapatite (HA) that is the major inorganic component of bones and constitutes approximately 70% of bone structure (Kattimani et al., 2016; Ramesh et al., 2018; Mondal and Pal, 2019; Bal et al., 2020). HA can be combined with various natural or synthetic polymers and/or growth factors to promote bone formation and regeneration by enhancing its conductivity and/or inductivity.

**FIGURE 4:**
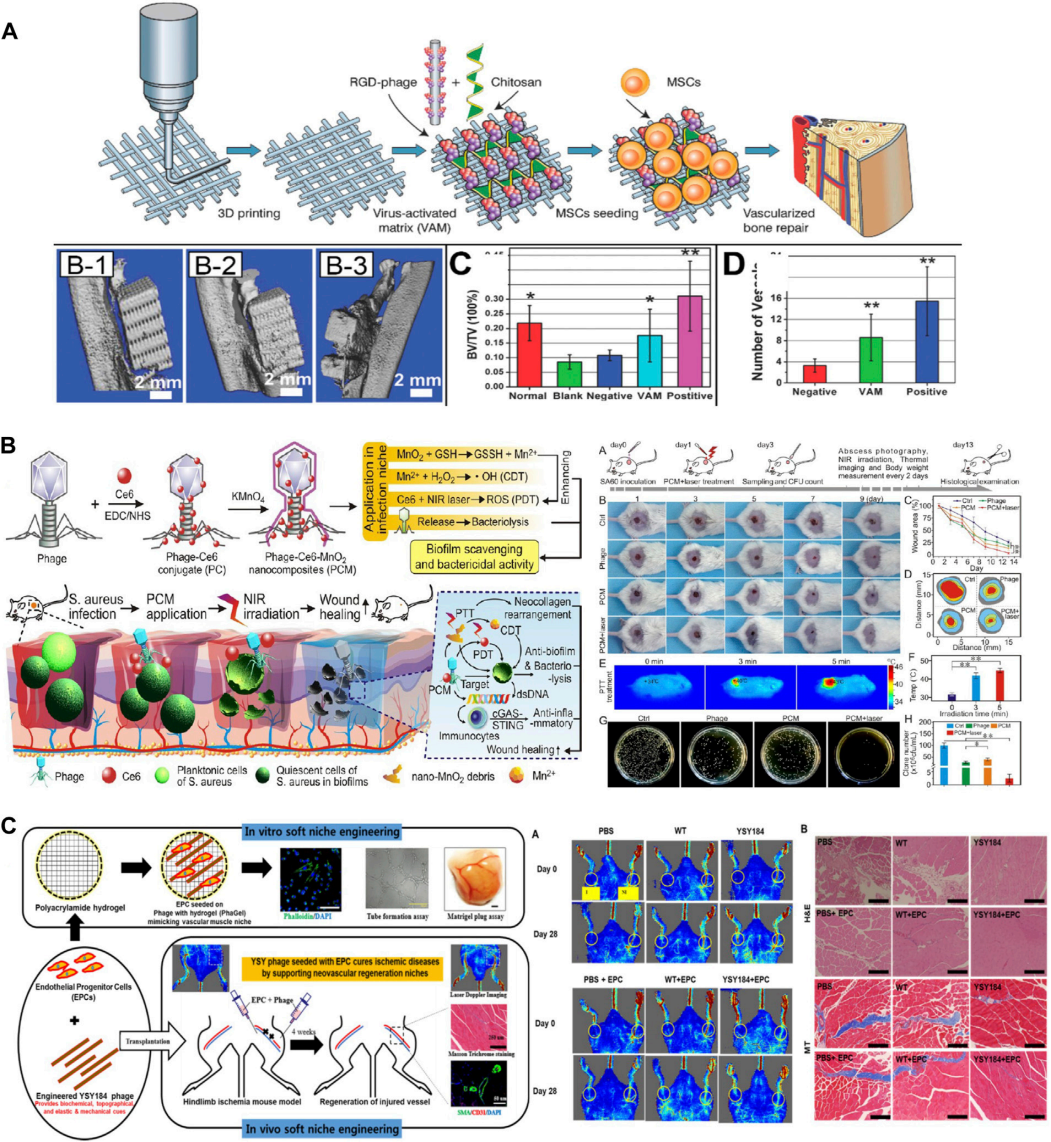
Application of phages in tissue regeneration. **(A)** Bone regeneration using an RGD-inserted M13 phage (Reprinted with permission from Cao et al., 2016. Copyright 2016; American Chemical Society). **(B)** Antibacterial and wound healing assays using phage-chlorin e6 (Ce6)-manganese dioxide nanocomposite (PCM) with exposure to the NIR laser (Reprinted with permission from Wang et al., 2023. Copyright 2023; American Chemical Society). **(C)** Angiogenesis at the ischemic site using a polyacrylamide (PA) hydrogel containing RGD-inserted M13 phages (Shrestha et al., 2022, Copyright 2022; Elsevier B.V.).

Engineered phages have also been used as biocompatible materials for bone regeneration. Many researchers have modified phages with functional peptides obtained through phage displays to increase their regeneration efficiency. Wang et al. reported that arginine-glycine-aspartic acid (RGD)-M13 induces vascularized osteogenesis in 3D scaffolds, including HA/β-TCP and chitosan, with MSCs (Figure 4A) (Wang et al., 2014; Cao et al., 2016; Staquicini et al., 2021). Similarly, to overcome the limitations of tissue regeneration using only the M13 phage, Lee et al., 2016 used alginate conjugated with RGD-modified M13 as a bioactive material in an electrospun scaffold using polycaprolactone (PCL) for bone regeneration. In this study, the PCL scaffold coated with alginate and the RDG-M13 phage exhibited the highest osteogenic effect. Ramaraju and Kohn synthesized a novel peptide (DPI-VTK) composed of a cell-specific peptide (DPIYALSWSGMA, DPI) and an apatite-specific peptide (VTKHLNQISQSY, VTK) that were both identified through phage display screening (Ramaraju and Kohn, 2019). This peptide catalyzed osteogenesis and vasculature formation equivalent to that of the clinical control using the collagen-based P15 peptide (GTPGPQGIAGQRGVV). Furthermore, Barros et al., 2020 developed a phage-loaded hydrogel for osteogenesis and anti-infection using alginate-nano HA. They used alginate-nano-HA to induce bone formation and loaded the vB_EfaS_LM99 (LM99) phage infecting *Enterococcus faecalis* to protect against bacterial contamination during implantation (Barros et al., 2020).

#### 3.1.3 Skin regeneration

The skin is a multifunctional organ that not only helps regulate body temperature and allows us to sense stimuli from the environment but also plays a crucial role in protecting us from potential harm by acting as a primary barrier against external threats. In cases where the skin is affected by inflammatory diseases, chronic conditions, or burn wounds, it becomes more susceptible to infection by various microorganisms, and this can lead to several types of damage such as discomfort, delayed healing, and complications (Shpichka et al., 2019). Although cell regeneration is crucial for the recovery of damaged skin, skin regeneration using phages has been demonstrated to be more effective. This is because phages possess antibacterial activities that make them highly efficient at combating bacterial infections. Moreover, phage-based skin regeneration helps to overcome antibacterial resistance (Gupta et al., 2019; Górski et al., 2020; Nguyen et al., 2021).

Jin and Lee proposed a method for promoting skin regeneration using fibroblasts (NHC-3T3) that are a key factor in this process (Jin and Lee, 2018). They fabricated a phage film using two different approaches that included drop-casting (isotropic) and shear-induced alignment (anisotropic) of phage matrices. These matrices were coat protein-engineered M13 phage that was treated on 3-aminopropyl triethoxysilane (APTES)-treated glass slide substrates. This study demonstrated that the phage film exhibited significant potential for fibroblast regeneration, ultimately resulting in accelerated skin regeneration.

Zhang et al., 2020 developed a hydrogel, named as ABgel, that includes biomaterials (such as hyaluronic acid, ox-alginate, and gelatin), bacteriophages, and growth factors (acidic fibroblast growth factor, aFGF) to enhance wound regeneration and combat antibiotic-resistant bacteria. In this study, we demonstrated that ABgel outperformed HOGgel (comprising only hyaluronic acid, oxalginate, and gelatin), Agel (HOGgel with aFGF), and Bgel (HOGgel with phage) in terms of its antibacterial, cell proliferation, and regeneration effects. This also indicates that when bacteriophages are used in combination with biomaterials on damaged tissue, it results in further enhanced regeneration due to the antibacterial properties of the phages.

The treatment of infected wounds using bacteriophages has recently been studied, and it encompasses not only genetic engineering but also chemical engineering by the conjugation of nanomaterials or chemicals. In a study conducted by Wang et al., 2023, they synthesized a phage (JD007)-Chlorin e6 (Ce6) conjugate (referred to as PC) via carbodiimide chemistry. Subsequently, they deposited MnO_2_ on the surface of PC, thus resulting in a nanocomposite known as the phage-Ce6-MnO_2_ nanocomposite (PCM). In a wound environment characterized by mild acidity and high glutathione (GSH) levels that are associated with infections caused by *Staphylococcus aureus*, the PCM was degraded into ultrasmall nanosheets (Figure 4B). When the nanomaterial was tuned to absorb near-infrared (NIR) wavelengths, it exhibited moderate photothermal therapy via Ce6 and chemodynamic therapy via the release of Mn^2+^. Additionally, photothermal therapy was performed using zinc ions (Zn^2+^) (Peng et al., 2022). A Pol-K peptide (GCFCEDACDKCG) that specifically attaches to Zn^2+^ was chemically conjugated with bacteriophage (M13-g3p (Pf1))-gold nanoparticles (AuNPs) to form a phanorod, and Zn^2+^ was then loaded onto the phanorod (phanorod-Zn). Photothermal therapy using NIR (808 nm laser) rapidly reduces *Pseudomonas aeruginosa* and releases Zn^2+^ to promote wound healing. These new approaches can overcome concerns regarding phage therapy such as instability, unsecured safety, and poor biofilm penetration.

#### 3.1.4 Other examples of regeneration

Nerve damage, particularly brain damage observed in diseases such as Alzheimer’s and Parkinson’s disease, can be highly harmful to humans. Zhou et al., 2019 developed a nano-to-micro hierarchical nanoridge-in-microridge (NiM) structure using the M13 phage. They achieved this with and without the genetic display of a foreign peptide (RGD) using the dip-pulling method. The structure of NiM was optimized by controlling the substrate pH and pulling speed. This phage structure could induce the bidirectional differentiation of human induced pluripotent stem cells (iPSC) into neurons and astrocytes. This co-differentiation promoted in the NiM structure exhibits the potential for use as a treatment method for neurodegenerative diseases.

Ischemic disease occurs when there is an inadequate blood supply and, consequently, insufficient oxygen delivery to specific tissues or organs, primarily the heart and brain (e.g., stroke or myocardial infarction). Shrestha *et al.* studied the curative effect of ECM-mimicking niches, including nanofibrous RGD-engineered M13 phages, in a polyacrylamide (PA) hydrogel (Figure 4C) (Shrestha et al., 2022). Endothelial progenitor cells (EPCs) and phages were injected into the ischemic area. This resulted in angiogenic (blood vessel formation) and antioxidant (protection against oxidative stress) functions by overcoming the pathological environment at the ischemic site.

#### 3.1.5 Limitation of phage as clinical materials

As described in this chapter, genetically engineered phages have been employed for tissue regeneration, such as skin, bone, and nerves. Many research groups have utilized genetically engineered phages in combination with biocompatible materials (e.g., HA, PCL) for tissue regeneration. Phages have shown significant potential for effective tissue regeneration. However, their applications as clinical materials are limited by several limitations. Phages are readily absorbed and have short half-lives *in vivo* due to rapid destruction by the immune system (Pirnay et al., 2018). In addition, there is a lack of accumulated research on the safety and stability of using phages as antibiotics or therapeutic agents for humans. Despite an increase in the number of clinical trials involving phages since 1990 (Strathdee et al., 2023), there are currently no FDA-approved phage therapies. The first clinical trial evaluating genetically modified phages was approved in the United States in 2022 (Yang et al., 2023). Accumulating safety and stability data through various clinical trials is essential to evaluate their suitability for human application, which will expand thier usability as clinical materials.

### 3.2 Phages for use as biosensors/sensing nanomaterials

The versatile M13 bacteriophage has been recognized as a useful material for biosensor fabrication due to its high tolerance to harsh conditions such as high pH, temperature, and ion concentration and also its cost-effective production, ease of fabrication and modification, and large density of binding sites (Tom et al., 2016; Moon and Park, 2017; Moon et al., 2019). In recent years, by leveraging these advantages of M13 phage researchers have developed various biosensors based on engineered M13 phages. These biosensors demonstrated high sensitivity and selectivity for detection, and their practical applicability was successfully demonstrated (Table 2).

**TABLE 2:** M13 bacteriophage-based biosensors.

Biosensor substrates	Detection target	Read-out method	LOD	Reference
**M13/AuNP**	Mercury	Color analysis	80 nM	Wang et al. (2017)
**M13/AgNP**	Mercury	Color/Optical analysis	10.8 nM	Manivannan et al. (2018)
**M13/Ag-coated AuNW**	Mercury	Color analysis	124 nM	Manivannan et al. (2020)
**M13/AuNP**	*E. coli*, *P. aeruginosa*, *V. cholera, X. campestris*	Color analysis	∼100 cells	Peng et al. (2019)
**M13/AuNP/MNP**	Avian influenza virus	Visual plaque counting	50 PFU/mL	Xu et al. (2021)
**M13/Au coated Si wafer**	Xylene, humidity	Color analysis	5 ppm xylene, 5%–90% humidity	Lee and Fan (2017)
**M13/Au coated Si wafer**	HEK-293, NCI-H1299, SK-Hep-1, HeLa, HCT116	Color analysis	n.a	Moon and Kim (2017)
**M13/Au coated Si wafer**	TNT, MNT, DNT	Color analysis	40 p.m. TNT, 400 uM MNT, DNT	Moon et al. (2018)
**M13/Au coated Si wafer**	Antibiotics	Color analysis	n.a	Moon and Park (2017)
**M13/Au coated Si wafer**	Estrogens, Antibiotics	Color analysis	n.a	Kim et al. (2020)
**M13/Si wafer**	Humidity	Color analysis	20%–90% humidity	Nguyen et al. (2021)
**M13/scFv**	MC38-CEA, CT26-CEA, human CEA	Optical/Surgical analysis	n.a	Murgas et al. (2018)
**M13/AgNW**	Pesticides (Paraquat)	SERS	40.2 ng/cm^2^	Koh et al. (2018)
**M13/AgNP**	*S. aureus*	SERS	10 cfu/mL	Wang et al. (2021)
**M13/PEDOT/Au-coated glass**	HSA	EIS	100 nM	Ogata et al. (2017)
**M13/CNF/GCE**	L-cysteine	EIS	20 uM	Szot-Karpińska et al. (2019)
**M13/AuNP/GCE**	*E. coli XL1*-Blue, *E. coli K12*	EIS	14 CFU/mL	Sedki et al. (2020)
**M13/Au, Ge-coated Si wafer**	Humidity, VOCs, EDCs	Color analysis	10 ppb VOCs, 150 ppb EDCs, 20%–90% humidity	Yoo, Kim, and Ko (2020)
**M13/AuNP/QD/GCE**	c-Met protein	EIS	1 pg	Pourakbari et al. (2022)

n.a., not available; MNP, magnetic nanoparticle; GCE, glassy carbon electrode.

#### 3.2.1 Advantages of phage in biosensors

M13 phage have been studied extensively in biosensor development among various phages. This phage has been mostly used as a scaffold for the immobilization of nanomaterials or as a particle to display target-specific ligands on its surface (Janczuk et al., 2016). The properties of M13 phage such as its highly engineerable nature, various functionalization sites for chemical reactions, and nontoxic self-assembly give advantages when we use M13 phage as a sensor material (Kao et al., 2023). Especially, the unique advantage of M13 phage over other biomaterials is that it can expose on its surface the ligands such as protein and peptide with binding affinity for target molecules. Their target-binding properties can be easily controlled through genetic or chemical modifications (Kim et al., 2016; Wang et al., 2023). Additionally, M13 phage can form a self-assembled structure that is distinguished from the other virus-based material. Phage-based self-assembly can be utilized as a variety of ordered nanomaterials through easy tunability of its bundles in sensor devices (Kim et al., 2023). There are also several drawbacks such as low transduction efficiency of phage and slower growth rate of engineered phage, however, these problems were partially improved through the control of transduction factors and the introduction of phagemid vector-based display system (Kim et al., 2016; Kao et al., 2023). Therefore, due to the multi-functionality and unique properties of M13 phage, M13 phage has been considered as a powerful candidate for target-specific and selective sensor applications (Table 3).

**TABLE 3:** Advantages and disadvantages of nanomaterials used in biosensors.

Nanomaterials	Advantages	Disadvantages	Reference
M13 Phage	• Large-scale and economic production	• Low transduction efficiency of phage	Kim et al. (2016), Cao et al. (2023), Wang and Li et al. (2023)
	• Great engineerable specificity		
	• High surface functionalization capability	• Slower growth rate of exogenous DNA fragment inserted phage	
	• Nontoxic self-assembly		
Dendrimers	• Small size	• Low aqueous solubility	Emencheta et al. (2024)
	• High functionalization capability	• Nonspecific toxicity	
	• High targeting potential	• Low scalability potential	
Lipid membrane	• Biocompatible structure	• Solvent residue can affect the mechanical properties of membrane	Ghazizadeh and Nasery (2023)
	• High sensitivity and selectivity	• Instability of solvent-free lipid membrane	
	• Small size	• Collapse by a mechanical or electrical shock	
Liposome	• Large surface area	• Thermodynamically unstable system	Mazur et al. (2017)
	• Non-toxic		
	• Biocompatible and biodegradable		
Carbon-based nanomaterial (carbon nanotube)	• Large surface area	• Nonspecific adsorption of protein	Cho et al. (2020)
	• Excellent conducting and electro-catalytic properties	• Formation of irreversible aggregates in aqueous solution	
		• Need to functionalize surface for increasing biocompatibility	
Carbon-based nanomaterial (graphene)	• Large surface area	• Hard to dissolve in water	Cho et al. (2020)
	• Fast electron transfer		
	• Ease of surface modification		
	• High thermal conductivity		

#### 3.2.2 Colorimetric sensors

Colorimetric sensors have attracted considerable attention in the context of M13 bacteriophage-based biosensors due to their advantages such as facile manufacturing processes and straightforward and rapid detection methods (Kim et al., 2020). Recently, diverse M13 bacteriophage-based colorimetric sensors have been investigated using phage display techniques to detect various molecules, including relative humidity, harmful chemicals, medical chemicals, and pathogens (Lee and Fan, 2017; Wang et al., 2017; Kim et al., 2020; Manivannan et al., 2020; Nguyen et al., 2021). Typically, in colorimetric sensors of this kind, metallic nanoparticles such as gold and silver serve as colorimetric probes for sensitive chemical detection. Conversely, engineered M13 bacteriophages function as specific targeting ligands for the selective detection of desired targets and also as templates for the *in situ* growth of nanoparticles. A functionalized M13 bacteriophage-based assay using networks between AuNPs and phages was constructed to sense mercury selectively and sensitively with a limit of detection (LOD) of 80 nM (Wang et al., 2017). Manivannan and colleagues developed two types of phage-based colorimetric sensors for mercury sensing using silver nanoparticles (AgNPs) and gold nanowires (AuNWs), respectively (Manivannan et al., 2018; 2020). The biosensor was constructed by conjugating the engineered M13 phage with AgNPs and demonstrated efficient metal ion detection with a low detection limit of 10.8 nM (Manivannan et al., 2018). Additionally, a colorimetric biosensor based on gold nanowires (AuNWs) was developed using M13 bacteriophages expressing gold-binding peptides (Manivannan et al., 2020). In particular, silver-coated M13 phage-AuNW complexes offer multiple Hg^2+^ binding sites with high flexibility, thus leading to superior sensing performance without aggregation in contrast to that provided by spherical Au nanoparticles (Manivannan et al., 2020).

**FIGURE 5:**
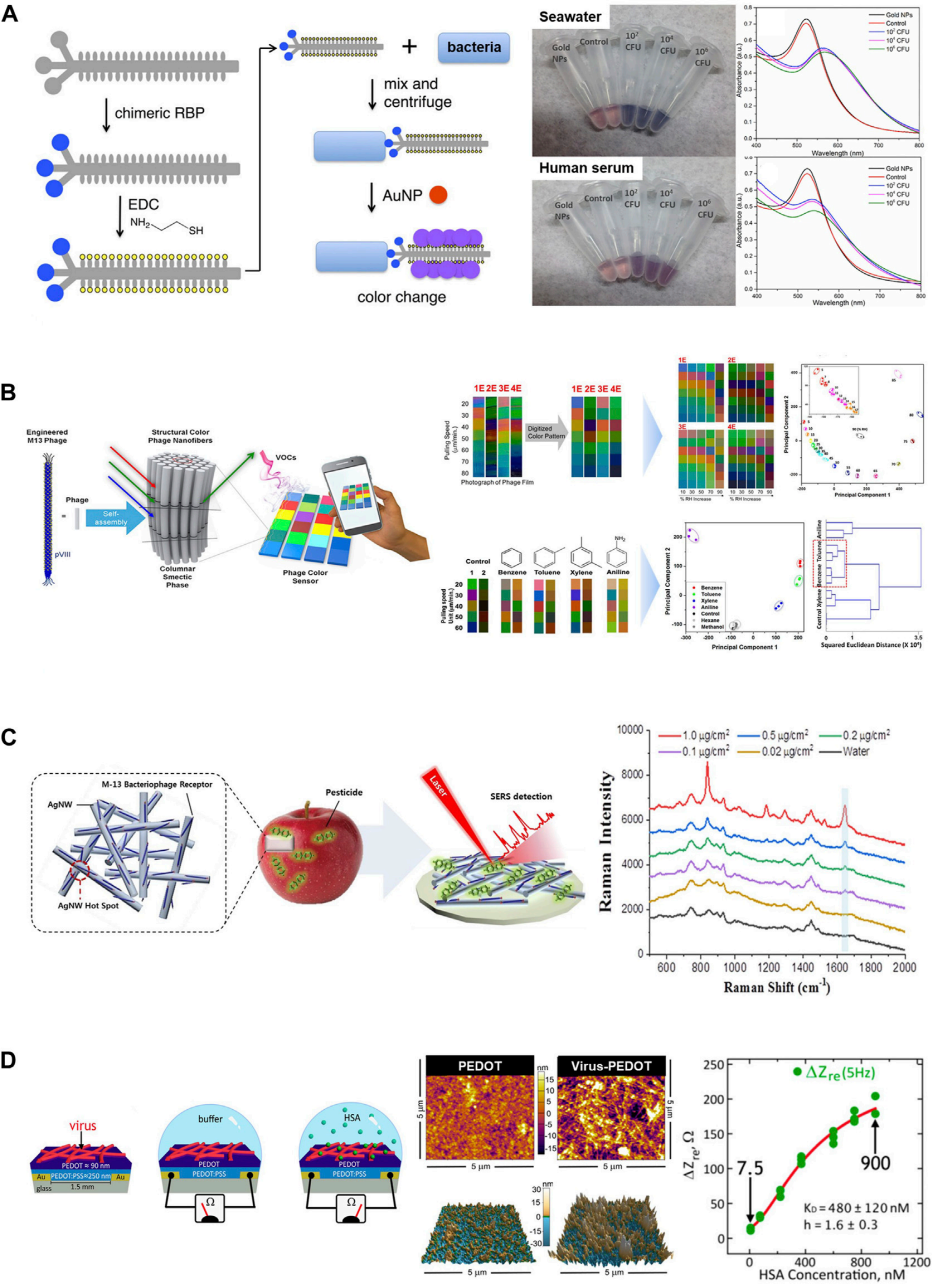
Application of M13 phages as biosensors. **(A)** The M13 phage-based colorimetric sensor for bacterial detection. Chimeric phages with bacteria-binding proteins and gold nanoparticle-binding motifs can detect bacterial species such as *E. coli* ER2738 in seawater or human serum (Reprinted with permission from Peng and Chen, 2019. Copyright 2019, American Chemical Society). **(B)** An M13 phage-based color sensor and multiple color arrays for sensing relative humidity and detecting benzene, toluene, xylene, and aniline (BTXA) (Reprinted with permission from Lee and Fan, 2017. Copyright 2017; American Chemical Society). **(C)** M13 phage-functionalized BPWHW/AgNW SERS sensor for detecting pesticides on the apple peel surface (Reprinted with permission from Koh et al., 2018. Copyright 2018; American Chemical Society). **(D)** Schematic diagram of the electrochemical biosensor using phage-PEDOT film for detecting the target HSA protein (left) and plan-view scanning electron microscope (SEM) images and atomic force microscopy (AFM) images of a PEDOT-only film and a phage-PEDOT film (middle). The plot demonstrates that the impedance was increased as the HSA concentration increased (right). (Reprinted with permission from Bhasin and Ogata et al., 2017. Copyright 2018, American Chemical Society).

A simple strategy was introduced that relies on the interactions between bacterial cells and modified M13 bacteriophage scaffolds for the rapid and sensitive detection of various pathogenic bacteria such as *E. coli*, the human pathogens *P. aeruginosa* and *Vibrio cholera*, and the plant pathogen *Xanthomonas campestris* (Figure 5A) (Peng and Chen, 2019). Thiolation of phages decorated with receptor-binding proteins intended to target various bacterial pathogens allows for the binding of AuNPs. This attachment results in the formation of aggregates on the phage surface. When exposed to pathogens, a color change was observed due to the aggregation of AuNPs. Furthermore, this assay can selectively detect bacterial species without any cross-reactivity while maintaining a low detection limit comparable to that of other assays (Miranda et al., 2011; Wang et al., 2015). It also demonstrates consistent performance across different media types, including tap water, seawater, and human serum, thus making it suitable for various environmental conditions.

In another study, researchers utilized a combination of magnetic nanoparticles and complexes formed by M13 phages and AuNPs for the visual quantification of the avian influenza virus (AIV) (Xu et al., 2021). They modified AuNPs with both antiphage and anti-AIV antibodies that were then bound to M13 phages, thus resulting in the formation of a phage-AuNP complex. Both probes, the phage-AuNP complex, and the modified magnetic nanoparticles simultaneously recognized the pathogen and formed relatively large complex particles compared to the individual gold and magnetic nanoparticles. By creating eye-visible and bright millimeter-sized plaques of these sandwich complexes on a plate, AIV can be directly detected at a sensitivity similar to that of digital polymerase chain reaction (dPCR) (Xu et al., 2021).

Moreover, upon exposure to the target materials, colorimetric sensors based on M13 phage bundles can exhibit distinct and visually detectable color changes due to the highly ordered and self-assembled nanostructure of the phage (Kim et al., 2020). Additionally, a pattern-recognizing multiarray sensor can be fabricated by integrating three types of chips composed of engineered M13 phages, including wild, RGD-modified, and EEEE-modified. This integrated-array sensor can be effectively applied to the classification and discrimination of target molecules, including antibiotics and hormonal drugs. This was achieved through an easy and rapid analysis of color-change patterns using hierarchical cluster analysis (Kim et al., 2020). A bundled phage nanostructure capable of forming different colored matrices on a single chip that varied according to the bundle diameter was prepared through a surfactant-assisted self-assembly process (Lee and Fan, 2017). A multiple-color array was constructed by introducing different engineered M13 phages of various diameters into the chip. It can be used to detect humidity and distinguish between harmful aromatic molecules that possess similar structures (Figure 5B) (Lee and Fan, 2017).

A phage-based electronic nose sensor consisting of multiple sets of functionalized phage bundles was developed to monitor cellular metabolism in the vapor state (Moon and Kim, 2017). This M13 phage-based nose sensor detected and identified several vapor-phase molecules produced in cells and cancer cells by analyzing color pattern changes according to the crystal structures of the phages (Moon and Kim, 2017). In additional studies, Moon and colleagues reported that the development of two types of structural color-based sensor utilizing engineered M13 phages (Moon and Park, 2017; Moon et al., 2018). Structural color matrices based on phages were constructed by simply controlling the pulling rate of the functionalized M13 phage bundles. This system exhibits noticeable color changes when exposed to organic solvents (Moon and Park, 2017), antibiotics, and harmful chemicals such as trinitrotoluene (TNT) (Moon et al., 2018). This suggests that it has the potential to be used as a platform for real-time environmental monitoring.

A programmable microcolor patterning technique utilizing the self-assembly of M13 phages was proposed as a practical alternative tool to overcome the limitations of existing self-assembly based micropatterning systems. These limitations include challenges associated with large-scale manufacturing due to multiple complicated processing steps and high production costs (Nguyen et al., 2021). To confirm the practicality of the developed technique for real-world use, barcode-like color patterns were fabricated by controlling the deposited thickness during color patterning on a SiO_2_ substrate. Microcolor patterns of M13 self-assemblies allowed for accurate detection of a wide range of humidity from 20% to 90% (Nguyen et al., 2021). As an additional and effective strategy for the large-area fabrication of sensors and to achieve rapid and sensitive detection of specific targets, a novel M13 phage-based colorimetric sensor using a highly lossy resonant promoter (HLRP) as the substrate has been suggested (Yoo et al., 2020). The ultrathin layer coated with the bundle of M13 phage exhibited enhanced colorimetric properties due to the resonance enhancement effect of the HLRP. The optimized sensor possessed the capability to successfully detect humidity over a broad range. Additionally, it can classify volatile organic chemicals (VOCs) at concentrations as low as 10 ppb, including acetone, isopropyl alcohol, diethyl ether, and benzene. This sensor can identify endocrine-disrupting chemicals (EDCs) such as diisobutyl phthalate (DiBP) and di-n-butyl phthalate (DnBP) at concentrations as low as 150 ppb.

M13 phages are widely used as valuable probes in biosensors to detect different biomarkers and diagnose various diseases. Genetically engineered M13 phage particles targeting the carcinoembryonic antigen (CEA) that is highly expressed in colorectal cancer cells were prepared and used for direct detection of tumor cells (Hammarström, 1999; Murgas et al., 2018). In this study, we demonstrated that the CEA-targeting M13 phage displayed specific binding to both purified CEA proteins and *in vitro* CEA-expressing tumor cells. Furthermore, tumor infiltration by neutrophils and macrophages was increased in samples treated with functionalized phages compared to levels in controls treated with phosphate-buffered saline (PBS) or wild-type M13 phages. These findings suggest the potential use of CEA-specific M13 phages in immunotherapy against colorectal cancer (Murgas et al., 2018). The M13 phage-based biosensor incorporating tungsten disulfide quantum dots and AuNPs demonstrated the ability to detect tyrosine–protein kinase Met (c-Met) protein, a biomarker for colon cancer, with an impressive limit of detection of 1 pg. This biosensor exhibited superior sensitivity in regard to detecting c-Met protein in clinical samples and surpassed the performance of common ELISA techniques (Pourakbari et al., 2022).

#### 3.2.3 Surface-enhanced Raman scattering

Raman signals can be greatly enhanced using surface-enhanced Raman scattering (SERS) technology that enables the ultrasensitive detection of target molecules (Park et al., 2015). Additionally, the SERS response can be amplified by introducing a functionalized M13 phage probe into an existing SERS biosensor system. This enhancement is achievable because phages can specifically bind to target molecules without substantially impacting the electromagnetic field enhancement between the metal nanostructures (Park et al., 2015; Koh et al., 2018; Wang et al., 2021). The feasibility of the M13 phage-functionalized silver nanowire (AgNW)-SERS sensor was demonstrated for the first time through the selective detection of pesticides, particularly paraquat (PQ) (Koh et al., 2018). The AgNWs were deposited onto a paper membrane, thus effectively forming high-density hotspots capable of enhancing the SERS signal. Subsequently, the engineered M13 phage that was decorated on the AgNW surface selectively and sensitively captured PQ (Figure 5C) (Koh et al., 2018). Another M13 phage-SERS biosensor was constructed to detect *S. aureus*. The M13 phage served as a SERS probe by displaying *S*. *aureus*-binding peptides, ultimately leading to higher selectivity and sensitivity of SERS signals (Wang et al., 2021). These studies demonstrate the possibility of the use of M13 phage-based SERS as a novel strategy for the enhancement of Raman signals and the sensitive detection of targets (Koh et al., 2018; Wang et al., 2021).

#### 3.2.4 Electrochemical biosensors

Phage-based electrochemical biosensors generate specific high-density binding signals for target proteins by circumventing the sources of noise in electrochemical biosensing. This capability enables the reproducible and sensitive detection of targets (Ogata et al., 2017; Bhasin et al., 2018). A phage-poly (3,4-ethylenedioxythiophene) (PEDOT) film was utilized as an electrode in a label-free electrochemical biosensor to directly sense target proteins such as human serum albumin (HSA) in aqueous buffer (Figure 5D) (Ogata et al., 2017; Bhasin et al., 2018). The Biosensor, comprising electrodes coated with M13 phage particles that incorporated HSA-binding peptides and was successfully recognized HSA in both PBS buffer and synthetic urine (Ogata et al., 2017).

M13 phage particles can also act as polyanions or polycations depending upon the pH of the solution. These properties enable nonspecific electrostatic interactions between phages and charged molecules (Avery et al., 2009). The use of M13 phage as a supporting material for the electrode of a biosensor for the detection of L-cysteine, a thiol biomarker, has been previously reported (Szot-Karpińska et al., 2019). The M13 phage-carbon nanofiber (CNFs)-based electrode was readily fabricated through non-specific electrostatic interactions between CNFs and wildtype phages and exhibited improved electrochemical properties compared to those of CNF-based electrodes (Szot-Karpińska et al., 2019). An M13 phage-based electrochemical impedance spectroscopy (EIS) cytosensor was proposed for the detection of fecal coliforms (Sedki et al., 2020). This biosensor successfully captured *E. coli species XL1*-Blue and *K12* strains with a remarkable limit of detection of 14 CFU/mL, and this is the lowest reported sensitivity to date among EIS-phage-based sensors. Additionally, the sensor retained its performance under harsh pH and temperature conditions for up to 2 weeks. It exhibited a sensitivity comparable to its initial performance in coliform detection tests using simulated river water, thus indicating its practical applicability for detecting coliforms in the field.

## 4 Challenges in genetically engineered phages

While engineered phages hold promise as novel nanomaterials with numerous advantages, their applications continue to present several technical and ethical challenges.

### 4.1 Technical challenges

One of major technical challenges for genetically engineered M13 phages as nanomaterials is the limitation in displaying amino acid sequences and sizes in M13 particles. To ensure functional M13 phages, expression and assembly within bacterial cells are required. Subsequently, they must not self-destruct and be secreted without harming host cells. This process place limits on the sequence diversity and length of peptides that can be displayed (Sattar et al., 2015; Tsedev et al., 2022). The restricted selection of peptides available for display reduces the effectiveness of phage-based applications.

Large-scale production of genetically engineered M13 phages is essential for their utilization as innovative nanomaterials and applications. While methods for production have been established and used on the laboratory production scale for many years (Sambrook and Green, 2012). These methods often prove impractical or unsuitable for scaling up production of phages. Therefore, large-scale production requires careful consideration of several factors.

Numerous techniques have been adopted for the expression and purification of M13, including *E. coli* infection in batch cultures, fed-batch cultures, and continuous fermenters (Kick et al., 2015; Pirnay et al., 2015; Schooley et al., 2017; Kolenda et al., 2022). The shift from small-scale cultures to large-scale production introduces challenges concerning the maintenance of consistent growth conditions, contamination control, and the assurance of uniform mixing and aeration in bioreactors.

The unique M13 growth mode, characterized by secretion from the host cells without lysis, offers significant advantages for purification. The presence of phages in the supernatant of growing host cells are relatively free from cellular contaminants such as intracellular proteins, genomic DNA, and RNA (Branston et al., 2012). Consequently, purification methods for M13 phages are relatively simpler compared to lytic phages. Purification techniques such as desalting column, ion exchange chromatography, and size-exclusion chromatography have been employed (Zakharova et al., 2005; Monjezi et al., 2010; Reitinger et al., 2012), with precipitation using polyethylene glycol (PEG) via ultracentrifugation being the most common (Branston et al., 2012; Lee and Fan, 2017). Despite not requiring particularly sophisticated instrument, PEG precipitation has inherent drawbacks, with the residual PEG content in M13 stocks being notably high (Branston et al., 2012). Moreover, this process may lead to the generation of viral stocks containing PEG residues, potentially hindering the interaction of M13 with other molecules (Branston et al., 2012).

### 4.2 Ethical considerations

Genetically engineered M13 phages have been utilized as nanomaterials in both of *in vitro* and *in vivo* animal experiments. However, their limited application in human is not solely constrained by technical considerations but also by ethical challenges. This arises from several aspects as following: 1) immunogenicity. Genetically engineered M13 phages have the potential to induce immune response in susceptible individuals due to their proteins or modified amino acids. Moreover, the repetitive structures of their coat proteins have been shown to possibly trigger immune responses (González-Mora et al., 2021; Venturini et al., 2022). Although the replication and production of M13 phages do not entail bacterial cell disruption, they are synthesized within *E. coli* cells. This suggests the possibility that lipopolysaccharide (LPS) is present in engineered M13 phages despite purification efforts. 2) Antibiotic resistance. Genetically engineered organisms, including M13 phages, sometimes carry antibiotic resistance genes as part of their genetic modification. It could contribute to the spread of antibiotic resistance, and this gene delivery make more difficult to treat bacterial infections. 3) Unknown safety issues. Genetically engineered M13 phages may pose unknown and unpredictable risks to human health. Extensive testing is usually performed before applications to human, but unexpected results may occur. In addition to introducing genetic modification to express novel properties, certain characteristics of phages can also be changed (Verheust et al., 2010). It is well-known that phages do not naturally infect eukaryotic cells. However, phages can be engineered to target specific eukaryotic cells, such as M13 phages carrying fibroblast growth factor (FGF) to invade mammalian cells *in vitro* (Larocca and Baird, 2001). It has been reported that they consequently fail to produce new viral particles and decompose inside the cells, significantly reducing the risks to humans (Larocca et al., 1998). Nonetheless, the risk may increase if the genetically engineered phage possesses dangerous characteristics.

The use of genetically engineered phages also raises ethical concerns regarding their release into the environment. Firstly, they have the potential to transmit genetic material, such as virulence factors and antimicrobial resistance genes, to other organisms (Johannessen et al., 2005). This can lead to genetic pollution as modified genes spread throughout ecosystems. The release of genetically engineered phages may also cause unintended ecological disruption as a result of potential interactions with native organisms in unexpected ways or transfer specific genes to bacteria in their surrounding environment (Johannessen et al., 2005; Verheust et al., 2010). Additionally, the long-term effects of releasing engineered organisms into the environment are often uncertain. It can be difficult to predict how these organisms will behave over time and what impact they will have on biodiversity and ecosystem stability.

Addressing these ethical issues requires careful consideration of the potential risks and benefits of using genetically engineered M13 phages is necessary. Robust regulatory oversight and stakeholder engagement are essential to ensure transparent decisions based on ethical principles.

## 5 Conclusion and future perspectives

This review focused on current developments in genetically engineered bacteriophages as nanomaterials for screening functional epitopes, tissue regeneration, and biosensors. We also illustrated successful applications of genetically engineered bacteriophages for tissue regeneration and biosensors. However, applying engineered bacteriophages to tissue regeneration has significant challenges to be addressed, such as stability and safety concerns. Particularly, it is essential to thoroughly understand how phages behave when they enter the human body. Continuous developments in the engineered phages should be achieved to overcome these limitations. The research on the side effects and toxicity has been conducted by exposing biocompatible molecules on the surface of bacteriophage. Recently, studies have been conducted on artificially producing virus-like particles using protein structure prediction programs based on protein structures. Research can be conducted using the programs to create engineered bacteriophage structures that maximize their advantages while minimizing their disadvantages. Because bacteriophages have excellent therapeutic payloads, high structural stability, and ease of conjugation of specific target molecules, bacteriophages have also been studied as drug delivery carriers. In addition to biomedical applications, the antimicrobial activity of bacteriophages is receiving significant attention in food processing, which requires pathogen-free meals and drug development industries to overcome the antimicrobial resistance crisis. Future relevant research will lead to engineered phages as valuable nanomaterials for food processing and biomedical applications.
